# Green Tea Gargling Effect on Cough & Hoarseness After Coronary Artery Bypass Graft

**DOI:** 10.5539/gjhs.v7n5p266

**Published:** 2015-03-16

**Authors:** Mohammad Reza Aryaeefar, Hedayat Jafari, Jamshid Yazdani-Charati, Aria Soleimani

**Affiliations:** 1 School of Nursing and Midwifery, Mazandaran University of Medical Science, Sari, Iran; 2 Department of Medical-Surgical Nursing, School of Nursing and Midwifery, Mazandaran University of Medical Sciences, Sari, Iran; 3 Department of Biostatistics, School of Health, Mazandaran University of Medical Sciences, Sari, Iran; 4 Department of Anesthesiology, School of Medicine, Mazandaran University of Medical Sciences, Sari, Iran

**Keywords:** cough, hoarseness, intubation, CABG

## Abstract

**Introduction:**

Endotracheal intubation is a method necessary for controlling and maintaining airway during general anesthesia. Cough and hoarseness are common complications after endotracheal intubation. Inflammation has an important role in postoperative cough and hoarseness outbreak. Also it has been stated that green tea has anti-inflammatory properties. Therefore, the current study has been conducted to investigate green tea gargling solution effect on cough and hoarseness after coronary artery bypass graft (CABG) surgery.

**Methods:**

In this single-blind, randomized, & controlled clinical trial, we enrolled 121 patients undergoing CABG admitted to the ICU. The intervention group participants were asked to gargle 30 cc of green tea solution. The control group patients gargled 30 cc of distilled water. An hour after extubation, the intervention group patients were asked to gargle 30 cc of green tea and the control group patients were required to gargle 30 cc of distilled water every 6 hour up to 24 hour (each patient for 4 times). Moreover, the cough and hoarseness questionnaire was also filled in 6, 12, and 24 hours after endotracheal extubation.

**Results:**

The results showed no significant differences among the patients in both groups regarding age, gender, body mass index, smoking history, and anesthesia duration. There was a significant difference between the two groups in terms of cough 12 hours after removing the endotracheal tube. At other times, there was no significant difference between the two groups considering cough and hoarseness.

**Conclusion:**

The present study results showed that green tea gargling does not decrease hoarseness. Though, cough occurrence was less in the intervention group than the other group.

## 1. Introduction

Coronary artery bypass graft (CABG) has been one of the most commonly performed cardiac surgeries over the past two decades and its goal is to relieve angina pain and preserve heart muscle’s function (Sadeghi et al., 2007). In order to perform open heart surgery, the patients received general anesthesia and subsequently were transferred to the ICU to recover from anesthesia (Nikravan & Shiri, 2009). Endotracheal intubation is a method necessary for controlling and maintaining airway during general anesthesia. Cough and hoarseness are common post-endotracheal intubation complications. Although these effects are small, they lead to frustration and illness after anesthesia (Honarmand & Safavi, 2009). These effects may be created by inflammation and airway mucus scratches due to manipulation (Shaaban & Kamal, 2012). Cough incidence after the endotracheal tube removal has been reported, in some sources up to 70% (Navarro et al., 2012). Moreover, hoarseness incidence in the first post-operative day ranged 16%–55% in some articles (Yamanaka et al., 2009). Surface scratch, inflammation, and necrosis are the most commonly experienced cases in larynx (Andrea et al., 2011). The endotracheal tube type and size (Sumathi et al., 2008), using lubricant, the surgery duration, severe airway suction (Khezri et al., 2011), and drugs used during anesthesia, all affect the incidence of cough and hoarseness due to endotracheal intubation (Al-Qahtani et al., 2005). There are several pharmacological and non-pharmacological methods to treat and improve these disorders (Sumathi et al., 2008). Treatments used for endotracheal intubation induced cough and hoarseness are beclomethazone inhalation and azoline sulfate gargling (Ogata et al., 2005), gargling aspirin and benzydamine hydrochloride (Agarwal et al., 2006), lidocaine gel and intravenous lidocaine (Al-Qahtani et al., 2005), lubricating the endotracheal tube (Blunt et al., 2001), and using dexamethasone during extubation (Matsumoto et al., 2011). Despite all the measures taken, such as prevention, treatment, or the attempt to reduce the complications of endotracheal intubation, it appears that this complication incidence is still high (Hong et al., 2008). Green tea has been used since 3000 BC and is still used (Nie et al., 2008). Its anti-inflammatory and anti-oxidant effects have been approved (Yaghmaei et al., 2009). Green tea contains caffeine, catechins, polyphenols, vitamins B, C, and E, flavonoids, glycoprotein, and carotenoids fibroids (Hong et al., 2008). Glycoproteins have various biological activities, such as anti-inflammatory properties (Matsumoto et al., 2011). Since one of the main complications following endotracheal intubation is inflammation (Kazemi & Amini, 2007) and anti-inflammatory medication has been effective in this respect, green tea can be used to reduce these symptoms (Park et al., 2011). Researchers believe that green tea is a natural substance, anti-inflammatory, and harmless and can reduce cough and hoarseness. Therefore, the current study has been conducted to investigate the effect of green tea gargling solution on post-CABG cough and hoarseness.

## 2. Methods

This was an experimental, single-blind, randomized, and controlled clinical trial. The present study was conducted on 121 patients undergoing coronary artery bypass surgery admitted to the intensive care unit (ICU) of Mazandaran University of Medical Sciences affiliated Heart Center located in the north of Iran. The initial selection of the patients was performed considering the participants’ eligibility and after obtaining the consent of the Ethics Committee of Mazandaran University of Medical Sciences with the number and date 9.7.2014. Inclusion criteria were the age range of 30-70 years old (Hung et al., 2011), no history of having cold or sore throat during the preceding week, and lack of drug addiction (Shaaban & Kamal, 2012). Exclusion criteria were intubation taking more than 30 seconds (Ghaleb et al., 2013), Mallampti score higher than 2, more than 1 attempt for intubation (Tabari et al., 2013), active airway infection, and the history of allergy to green tea (Ghaleb et al., 2013). Furthermore, the patients kept in the ICU with an endotracheal tube for more than 16 hours, and those patients unable to communicate due to different reasons, such as dangerous dysrhythmias, bleeding, loss of consciousness, or any other ones, were excluded from the study (Emami Zeydi et al,. 2011). Based on the pilot study with variables of δ_1_ = 1.3 and δ_2_ = 2.37, and considering exclusion probabilities, sample size was calculated at 65 per group.


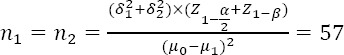


Thus, 130 patients enrolled in the study were randomized to experimental and control groups using random numbers table. After explaining the project to the eligible patients and obtaining written consent from them, checklist No.1 including demographic and medical information was completed in both groups in the operating room before anesthesia induction. All the patients underwent the same anesthesia method and similar drugs were used. For all the patients, endotracheal tubes from Supa Company (Tehran, Iran) were applied. For the male patients, tracheal tubes No. 7 and 6.5 were used, and for the female patients, tracheal tubes No. 6 and 6.5 were used. During the surgery and in the ICU, endotracheal tube cuff pressure was kept at 10 to 20 cm of water. During the airway suctioning surgery, straining, coughing, and existence of blood in the patient’s mouth and endotracheal intubation were recorded. After surgery, the patients were transferred to the ICU, and monitored. Within 4 to 16 hours after arriving at the ICU, the endotracheal intubation was removed according to the desired items under the supervision of an anesthesiologist physician. For all the patients, the same method of endotracheal tube removal was used. The Questionnaire of Coughing and Hoarseness was completed 1 hour after the endotracheal tube removal and ensuring the patients’ ability to communicate. To assess cough and hoarseness, a 4-point tool was used (Table 1). This questionnaires has been frequently used by previous researchers with similar subjects and its reliability and validity have been confirmed by test-retest (r = 0.6) and Mann-Whitney test (p ≤ 0.0004) (Shaaban & Kamal, 2012; Navarro et al, 2012; Sumathi et al., 2008).

**Table 1:** Scoring System for cough and Hoarseness

	Cough
No sore throat	0
Mild (less than a common cold)	1
Moderate (similar to a common cold)	2
Severe (more than a common cold)	3
	Hoarseness
No hoarseness	0
Mild (no hoarseness at the time of the interview but had it previously)	1
Moderate (is only perceived by the patient)	2
Severe (recognizable at the time of interview)	3

The intervention group participants were asked to gargle 30 cc green tea solutions at room temperature. The control group patients gargled 30 cc distilled water at room temperature. Green tea solution in the intervention group and distilled water in the control group were gargled every 6 hour after removing the endotracheal tube for 24 hours (4 times for each patient). During the intervention, the Cough and Hoarseness Questionnaire was filled in by a nurse unaware of the case and control groups 1, 6, 12, and 24 hours after the endotracheal tube was removed. Notably, the first time for completing the questionnaires was 1 hour after tracheal tube removal and before gargling green tea and distilled water. The objective was comparing the two groups regarding throat pain at baseline before any intervention.

In this study, descriptive and inferential statistics were used to analyze the data. Descriptive statistics (frequency distribution table, mean, and standard deviation) were employed for demographic characteristics. For comparing cough and hoarseness at different times in the two groups, repeated measures analysis of variance, Student’s t-test, and chi-square test were applied. Data analysis was performed using SPSS 16 for Windows (SPSS Inc., Chicago, IL, USA). The Cochran-Armitage test based results for the linear process of illness severity in the two independent groups of control and experimental were calculated by Winpepi software (version 2).

## 3. Results

In the present study, out of a total of 130 patients, 9 patients were excluded (for 5 patients intubation was performed twice, 2 patients had Mallampti score of higher than 2, the endotracheal tube was removed for 1 patient in the ICU, and 1 patient was unconscious over 16 hours). Of the 121 patients remaining in the study, 58 patients were placed in the intervention group and 63 patients in the control group. Of these patients, 62 were male and 59 were female. The patients’ mean age was 58.03. The results showed no significant differences among the patients in both groups regarding age, gender, body mass index, smoking history, and anesthesia duration (Table 2).

**Table 2:** Comparison between the two groups in terms of demographic and clinical information

	Intervention group (n = 53)		Control group (n = 63)		P-value
Gender	Male	29	Male	33	0.794
	Female	29	Female	30	
Age	57.7 ± 6.4		58.2 ± 7.8		0.688
Intubation duration	11.3 ± 3.06		11.4 ± 4.1		0.825
BMI	26.4 ± 3.4		26.3 ± 3.4		0.897
Smoking	14.44		16.47		0.813
History of diabetes	14.44		15.48		0.966

BMI: Body mass index.

In terms of cough (p = 0.707) and hoarseness (p = 0.536) before the intervention, no meaningful differences were observed between the two groups. This result was also seen 6 hours after the endotracheal tube removal. In terms of hoarseness, no significant differences were observed between the two groups and 12 hours after the endotracheal tube was removed. However, related to cough, there was a significant difference between the two groups (P=0.030). 24 hours after removing the endotracheal tube, no significant difference was seen between the two groups regarding cough and hoarseness (p = 0.221) and (p = 0.739) (Table 3).

**Table 3:** Sore throat and Hoarseness results at different times

After the surgery		0		6		12		24	
Co=Control In=Intervention		*Co n (%)*	*In n (%)*	*Co n (%)*	*In n (%)*	*Co n (%)*	*In n (%)*	*Co n (%)*	In n (%)
**Cough**	**No**	22 (34.9)	20 (34.4)	16 (25.4)	22 (37.9)	23 (36.5)	31 (53.4)	39 (61.9)	41 (70.7)
	**Mild**	26 (41.2)	24 (41.3)	27 (42.9)	20 (34.5)	30 (47.6)	23 (39.7)	19 (30.2)	15 (25.9)
	**Moderate**	13 (20.6)	13 (22.4)	18 (28.6)	15 (25.9)	9 (14.3)	4 (6.9)	5 (7.9)	2 (3.4)
	**Severe**	2 (3.1)	1 (1.7)	2 (3.2)	1 (1.7)	1 (1.6)	0 (0)	0 (0)	0 (0)
	**Total**	41 (65)	38(65.4)	47 (74)	36 (62)	40 (65)	27 (46)	24 (39)	17 (29)

**p value**		**0.707**		**0.229**		**0.030**		**0.221**	
**Hoarseness**	No	30 (47.6)	29 (50)	22 (34.9)	21 (36.2)	30 (47.6)	28 (48.3)	40 (63.5)	41 (70.7)
	**Mild**	23 (36.5)	21 (36.2)	23 (36.5)	23 (39.7)	31 (49.2)	26 (44.8)	18 (30.2)	15 (25.9)
	**Moderate**	9(14.2)	6 (10)	18 (26.8)	12 (20.7)	2 (3.2)	4 (6.9)	5 (7.9)	2 (3.4)
	**Severe**	1 (1.6)	2 (3.3)	0 (0)	2 (3.4)	0 (0)	0 (0)	0 (0)	0 (0)
	**Total**	35 (52)	29 (50)	41 (65)	37 (63)	33 (52)	30 (51)	24 (38)	17 (29)

**p value**		**0.536**		**0.878**		**0.774**		**0.739**	

## 4. Discussion

This study investigated the effects of green tea gargling on cough and hoarseness after intubation in patients undergoing CABG. In this study, 65% of the patients were coughing 1 hour after removing the endotracheal tube, and 52% reported symptoms of hoarseness. Navarro et al., examined the effect of filling the endotracheal tube cuff with lidocaine on cough and hoarseness, and found that about 80% of the patients were coughing during recovery and 40% of the patients complained about hoarseness. Perhaps the high incidence of cough in the patients was related to the fact that the participants entering the study were smokers (Navarro et al., 2012). Another study conducted by Sumathi et al., reported the incidence of cough up to 1 hour after removing the endotracheal tube as 50% and hoarseness as 45% consistent with the present study results (Sumathi et al., 2008). The results indicated that the incidence of cough 12 hours after surgery had decreased in the group that gargled green tea and had a significant difference. The incidence of cough decreased 24 hours after the endotracheal tube removal but was not statistically significant. In this study, no difference was observed between the groups at any incidence times or in the severity of hoarseness, meaning that gargling green tea does not decrease hoarseness. In the study that compared the effect of betamethasone gel and lidocaine gel on post-operative cough and hoarseness of endotracheal tube cuff, it was concluded that betamethasone gel affects post-operative cough and hoarseness but lidocaine gel does not (Sumathi et al., 2008). In the study investigating the effect of betamethasone gel and gargling ketamine, it was found that both medications reduced the symptoms but betamethasone gel was more effective at all. Since inflammation causes symptoms, such as coughing and hoarseness, it appears that anti-inflammatory drugs were more effective than the other drugs (Shaaban & Kamal, 2012). In another research studying the effect of inhaled fluticasone on cough and hoarseness after surgery, it was discovered that this procedure reduced cough and hoarseness after surgery. This study result regarding hoarseness was not compatible with that of the previous one (Tazeh-Kand et al., 2010). Tavakol et al., studied the effect of intratracheal lidocaine on the incidence of coughing after the endotracheal tube removal and concluded that it can reduce the incidence of cough (Tavakkol & Ghaffarian, 2009). This is probably because lidocaine causes numbness in the tracheal area and this decreases coughing. In the present study, after 24 hours, neither of the groups had severe cough and hoarseness. This suggested that the inflammation reduced over time without intervention. Nevertheless, gargling green tea reduces coughing due to a combination of glycoproteins and catechins present in green tea. Those who use green tea gargling reported fewer coughs than the other groups. Catechins are a group of polyphenols. So far 4 major groups of catechins have been identified in green tea leaves including epigallocatechin gallate (EGCG), epicatechin gallate (ECG), epigallocatechin (EGC), and epicatechin (EC). EGCG is a major component of green tea polyphenols and has anti-inflammatory properties (Matsumoto et al., 2011). In the studies performed on the complications of intubation, more focus was on the work technique and using a variety of different drugs such as local anesthetics or anti-inflammatory medications. It seems that using green tea, which is beneficial and safe, can be useful in this respect. The limitation of this study was that the patients gargling green tea were aware of its flavor and color and this issue may have affected the results. It is suggested that the effect of green tea gargling also be investigated on other groups of patients undergoing surgery because patients undergoing CABG have more complex problems and pain compared to the other surgeries.

## 5. Conclusion

The results of this study showed that green tea gargling does not decrease hoarseness. Although in the intervention group, cough occurrence was fewer than the other groups.
